# Self-rated health and its association with mortality in older adults in China, India and Latin America—a 10/66 Dementia Research Group study

**DOI:** 10.1093/ageing/afx126

**Published:** 2017-07-18

**Authors:** Hanna Falk, Ingmar Skoog, Lena Johansson, Maëlenn Guerchet, Rosie Mayston, Helena Hörder, Martin Prince, A Matthew Prina

**Affiliations:** 1 Institute of Neuroscience and Physiology, Neuropsychiatric Epidemiology, Sahlgrenska Academy, University of Gothenburg, Wallinsgatan 6, SE-431 41 Mölndal, Sweden; 2 Sahlgrenska Academy, Center for Ageing and Health—AGECAP, Gothenburg University, Wallinsgatan 6, SE-431 41 Mölndal, Sweden; 3 Health Services and Population Research Department, Psychology and Neuroscience, Centre for Global Mental Health, Institute of Psychiatry, King's College London, Strand, London WC2R2LS, UK

**Keywords:** Older people, self-rated health, mortality, low- and middle-income countries, 10/66 Dementia Research Group

## Abstract

**Background:**

empirical evidence from high-income countries suggests that self-rated health (SRH) is useful as a brief and simple outcome measure in public health research. However, in many low- and middle-income countries (LMIC) there is a lack of evaluation and the cross-cultural validity of SRH remains largely untested. This study aims to explore the prevalence of SRH and its association with mortality in older adults in LMIC in order to cross-culturally validate the construct of SRH.

**Methods:**

population-based cohort studies including 16,940 persons aged ≥65 years in China, India, Cuba, Dominican Republic, Peru, Venezuela, Mexico and Puerto Rico in 2003. SRH was assessed by asking ‘how do you rate your overall health in the past 30 days’ with responses ranging from excellent to poor. Covariates included socio-demographic characteristics, use of health services and health factors. Mortality was ascertained through a screening of all respondents until 2007.

**Results:**

the prevalence of good SRH was higher in urban compared to rural sites, except in China. Men reported higher SRH than women, and depression had the largest negative impact on SRH in all sites. Without adjustment, those with poor SRH showed a 142% increase risk of dying within 4 years compared to those with moderate SRH. After adjusting for all covariates, those with poor SRH still showed a 43% increased risk.

**Conclusion:**

our findings support the use of SRH as a simple measure in survey settings to identify vulnerable groups and evaluate health interventions in resource-scares settings.

## Background

Population-based studies in high-income countries have reported a relationship between older adults’ subjective perception of their overall health and outcomes such as functional performance, physical activity and morbidity [1, 2]. While self-rated health (SRH) is a subjective indicator of health status, it has been found to be a strong predictor of mortality [3, 4], as it integrates biological, mental, social and functional aspects of a person, including individual and cultural beliefs and health behaviours [5]. It is an all-inclusive, sensitive, non-specific measure that assesses health and predicts health outcomes in ways that are still unclear, and not necessarily identical with objective health status [6]. Lay definitions of health take a wide range of factors into account, and previous and present health experiences are likely to influence both what the person review as potential components of health and the way in which they are acknowledged [7, 8]. Constituent parts of health known to influence SRH, and subsequent mortality, include chronic illness, depression, cognitive function, socioeconomic status, functional impairment and physical activity [9, 2]. Studies show that SRH demonstrates an ability to identify groups with high future health service use and costs [10, 11], and that changes in SRH within the same individual over time may be based on comparisons with the person's own past health rather than comparisons of one's self to same aged peers [3].

In spite of strong empirical evidence from high-income countries, few studies to date have examined the association between SRH and mortality in older adults in low- and middle-income countries (LMIC). Because of the subjective nature of the indicator, transferability of empirical evidence, and validity of SRH across cultures is questionable [12], since context is likely to influence how people evaluate their own health. Nonetheless, findings from a small number of existing studies on the prevalence of SRH and its predictive value on mortality and morbidity in LMIC settings appear to be fairly consistent. Among older adults in China, poor SRH was positively associated with mortality, cardiovascular disease, cancer and respiratory disease [13], and childhood socioeconomic conditions exerted long-term effects on SRH and mortality, independently of adult and community socioeconomic conditions [14]. In India, males with poor SRH had a significant increase in mortality hazard, and lack of spousal support and disability significantly increased this hazard [15]. In Brazil, older adults with poor SRH had a 30% increased 10-year mortality risk compared to persons with good SRH, and the prevalence of good SRH was associated with male sex, more than 5 years of schooling, fewer diseases [16] and higher household income [17].

Many LMIC are currently undergoing rapid demographic, social and health transitions were the majority of older adults are outside the social safety net, posing a challenge to already overburdened societal systems [18]. Strong empirical evidence suggests that SRH may be useful as a brief and simple measure in the context of public health research, and with practical utility such as identifying vulnerable groups in resource-scarce settings for targeted health interventions [15, 19]. However, older adults’ assessment of SRH is directly contingent on their sociocultural context. It is important, therefore, to understand the meaning and impact of SRH cross-culturally. Although the 10/66 Dementia Research Group's (10/66 DRG) population-based studies of ageing and dementia has already explored the predictive validity of dementia [20], frailty [21], social networks [22] and chronic diseases [23], to our knowledge the predictive validity of SRH has never been assessed across a large group of LMIC. This study therefore aims to explore the prevalence of SRH and its association with mortality in older adults from China, India and Latin America in order to cross-culturally validate the construct of SRH.

## Method

### Setting and study design

The 10/66 Dementia Research Group's (10/66 DRG) population-based studies of ageing and dementia in LMIC comprise baseline surveys of all older adults, aged ≥65 years, living in 11 geographically defined urban and rural catchment area sites in eight LMIC (see Supplementary data, Appendix 1, available at *Age and Ageing* online). The current secondary analyses include data from urban and rural sites in China, Mexico, Peru and India, and urban sites in Cuba, Dominican Republic, Venezuela and Puerto Rico. Baseline population-based surveys were carried out between 2003 and 2007, and incidence wave follow-up assessments between 2008 and 2010. The design of the 10/66 DRG research program has been described in detail elsewhere [24, 25]. Here, we will describe aspects directly relevant to the analyses presented in this paper. All study instruments were translated, back translated by local investigators fluent in English and local languages and assessed for acceptance and conceptual equivalence [26]. All participants gave written informed consent. Local ethical committees and the ethical committee of the Institute of Psychiatry, King's College London, approved the study.

## Measures

### Self-rated health

The question concerning SRH was, ‘How do you rate your overall health in the past 30 days?’ with response options ranging from ‘very good’, ‘good’, ‘fair’, ‘poor’, to ‘very poor’. For the statistical analyses, these were combined into three categories; ‘good’ (including very good), ‘moderate’ and ‘poor’ (including very poor).

### Outcome—Mortality

Mortality was ascertained through a screening of all respondents in the follow-up phase of the study. A verbal autopsy interview with a suitable key informant was completed to ascertain the cause of death. Date of death was also recorded.

### Covariates—Socio-demographic characteristics and use of community health services

Information on age, sex, educational level, use of community health services (i.e. primary care, hospital services, private doctors and traditional healers) during the last 3 months, and number of household assets was collected using a standard socio-demographic questionnaire.

### Covariates—Health factors

Health conditions diagnosed by a physician were self-reported. In the present study, we used the following health conditions in our analysis; dementia, stroke, hypertension, chronic obstructive pulmonary disease (COPD), diabetes, depression and anxiety. Dementia diagnosis was made according to 10/66 criteria [26]. Systolic and diastolic resting blood pressure was measured in all respondents. Hypertension was ascertained according to the European Society of Hypertension criteria and/or a positive answer to the question ‘have you ever been told by a doctor that you have hypertension?’ COPD was diagnosed in those who responded ‘yes’ to the questions ‘do you usually cough up phlegm from your chest first thing in the morning?’ and ‘for how many months of the year does this usually happen?’ was 3 months or more. Diabetes and stroke were ascertained by a positive answer to the question ‘have you ever been told by a doctor that you have diabetes/stroke?’ Depression was ascertained according to the EURO-D [27]. Those scoring 4 or more on the scale was subsequently described as cases of EURO-D depression [28]. Anxiety was assessed using the Geriatric Mental State Examination [29], together with its diagnostic algorithm, the Automated Geriatric Examination for Computer Assisted Taxonomy (AGECAT). Respondents who reached level three in the GMS/AGECAT stage I anxiety axis were considered cases, as this threshold normally reflects a severity that warrants professional intervention [30]. Disability was assessed using the 12-item version of the WHODAS, a culture-fair assessment of difficulties during the past 30 days due to diseases or illnesses, other health problems that may be short or long lasting, injuries, mental or emotional problems, and problems with alcohol or drugs [30, 31].

## Analysis

All data were double entered into EPIDATA software and data analyses were performed using STATA version 14. For this study, we used the 10/66 data archive (release 3.4; March 2015). We reported the prevalence of SRH by sex and age group by study site, generating robust standard errors and 95% confidence intervals (CI). We used standardisation to compare the prevalence of SRH among the sites having adjusted for the compositional effects of age, sex and educational level (direct standardisation with the whole sample as the standard population). We used Poisson regression analysis to describe the association between SRH, socio-demographics and health factors across sites. We fitted the models separately for each site and used a fixed effects meta-analysis to combine them, estimating the degree of heterogeneity using Higgins’ *I*^2^. Cox proportional hazards models were then applied to examine the associations between ‘poor’ and ‘good’ SRH with mortality using ‘moderate’ SRH as reference (OR = 1.0) with a step-wise adjustment for all covariates. Again, we fitted the models separately for each site and then used a fixed effects meta-analysis to combine them, along with Higgins’ *I*^2^.

## Results

### Sample characteristics

In total, 16,940 persons aged ≥65 years participated in the study at baseline. Response proportions varied between 72% and 98% across sites (mean response rate 86%). Median years of follow-up was 4 years (range 2–5 years). Socio-demographic characteristics by study site are presented in Supplementary Table 1, available at *Age and Ageing* online.

### Prevalence of SRH

Table 1 shows the prevalence of SRH combined into three categories by sex and site. With the exception of China, SRH was higher in urban than rural sites. The highest prevalence of good SRH was found in urban India (76.8%, 95% CI 73.7–80.0%), followed by rural China (62.1%, 95% CI 59.4–64.9%), and Venezuela (57.5%, 95% CI 54.9–60.2%). The highest prevalence of poor SRH was found in Cuba (9.8%, 95% CI 8.2–11.3%), followed by Dominican Republic (8.8%, 95% CI 7.4–10.3%), and rural Mexico (8.7%, 95% CI 6.1–11.4%). Supplementary Table 2, available at *Age and Ageing* online, shows the prevalence ratios of SRH by site with the five levels of SRH as measured in the original survey. Supplementary Table 3, available at *Age and Ageing* online, shows the prevalence of SRH by age group and site. Table 2 shows the mutually adjusted prevalence rations from a Poisson regression analysis describing the association between poor SRH and socio-demographic factors and health factors.

**Table 1.** Prevalence ratios (95% CI) of SRH by gender and site

	Men	Women	Crude prevalence (95% CI)	Standardised prevalence (95% CI)^a^
Cuba				
Good	62.16 (59.14–65.09)	47.82 (45.55–50.11)	52.84 (51.01–54.67)	51.07 (48.39–53.75)
Moderate	31.03 (28.26–33.95)	41.64 (39.43–43.87)	37.92 (36.18–39.70)	39.15 (36.5–41.81)
Poor	6.81 (5.42–8.52)	10.54 (9.23–12.01)	9.23 (8.23–10.34)	9.77 (8.20–11.33)
Dom Rep				
Good	56.22 (52.46–59.91)	44.23 (41.54–46.95)	48.33 (46.09–50.58)	51.93 (49.35–54.50)
Moderate	35.43 (31.90–39.13)	46.04 (43.34–48.75)	42.41 (40.21–44.64)	39.00 (36.50–41.52)
Poor	8.35 (6.49–10.67)	9.74 (8.24–11.46)	9.26 (8.06–10.62)	8.84 (7.39–10.29)
Peru (U)				
Good	60.74 (56.41–64.90)	53.45 (50.11–56.75)	56.04 (53.33–58.71)	47.60 (43.63–51.57)
Moderate	34.36 (30.35–38.60)	41.36 (38.10–44.69)	38.86 (36.26–41.54)	44.85 (40.91–48.79)
Poor	4.91 (3.32–7.21)	5.20 (3.91–6.87)	5.09 (4.04–6.41)	7.09 (4.59–9.59)
Peru (R)				
Good	56.03 (49.89–61.99)	61.90 (56.22–67.29)	59.17 (54.82–63.37)	56.91 (52.83–60.99)
Moderate	42.41 (36.50–48.55)	35.71 (30.44–41.36)	38.84 (34.68–43.16)	38.52 (34.45–42.59)
Poor	1.56 (0.59–4.07)	2.38 (1.14–4.91)	2.00 (1.11–3.57)	2.00 (0.81–3.20)
Venezuela				
Good	66.38 (62.82–69.77)	54.97 (52.14–57.77)	59.15 (56.84–61.41)	57.50 (54.87–60.13)
Moderate	30.34 (27.06–33.84)	39.77 (37.04–42.56)	36.32 (34.13–38.57)	36.51 (33.94–39.08)
Poor	3.28 (2.19–4.88)	5.26 (4.14–6.66)	4.53 (3.68–5.57)	4.99 (3.89–6.07)
Mexico (U)				
Good	55.49 (50.15–60.71)	49.92 (46.14–53.71)	51.80 (48.72–54.86)	52.79 (49.68–55.90)
Moderate	37.39 (32.35–42.71)	40.60 (36.92–44.39)	39.52 (36.55–42.57)	37.78 (34.74–40.82)
Poor	7.12 (4.83–10.39)	9.47 (7.47–11.94)	8.68 (7.05–10.65)	8.26 (6.53–9.99)
Mexico (R)				
Good	54.52 (49.56–59.40)	49.00 (45.01–53.01)	51.20 (48.05–54.34)	47.80 (44.01–51.61)
Moderate	37.69 (33.02–42.60)	42.19 (38.28–46.21)	40.40 (37.30–43.58)	37.18 (33.16–41.20)
Poor	7.79 (5.53–10.87)	8.80 (6.79–11.34)	8.40 (6.84–10.27)	8.73 (6.10–11.37)
China (U)				
Good	14.83 (11.98–18.22)	15.43 (12.87–18.39)	15.17 (13.07–17.54)	15.57 (13.27–17.88)
Moderate	82.16 (78.56–85.27)	80.48 (77.29–83.32)	81.21 (78.70–83.48)	79.45 (76.98–81.92)
Poor	3.01 (1.82–4.93)	4.08 (2.82–5.88)	3.62 (2.70–4.85)	3.96 (2.88–5.03)
China (R)				
Good	67.26 (62.74–71.49)	70.14 (66.22–73.80)	68.66 (65.67–71.88)	62.12 (59.37–64.88)
Moderate	27.35 (23.42–31.67)	26.62 (23.13–30.43)	26.95 (24.11–29.98)	17.17 (14.65–19.69)
Poor	5.38 (3.63–7.90)	3.24 (2.05–5.07)	4.19 (3.12–5.60)	3.55 (2.13–4.98)
India (U)				
Good	84.01 (80.17–87.22)	73.91 (70.12–77.37)	78.27 (75.59–80.72)	76.82 (73.66–79.98)
Moderate	12.89 (10.00–16.45)	20.49 (17.36–24.03)	17.15 (14.94–19.61)	17.01 (14.33–19.69)
Poor	3.10 (1.81–5.27)	5.60 (3.99–7.82)	4.59 (3.45–6.07)	4.62 (2.75–6.50)
India (R)				
Good	29.52 (25.52–33.86)	22.02 (18.75–25.67)	25.43 (22.79–28.25)	28.69 (24.80–32.58)
Moderate	61.01 (56.44–65.40)	68.07 (64.05–71.84)	64.86 (61.88–67.74)	46.62 (42.69–50.56)
Poor	9.47 (7.10–12.52)	9.91 (7.66–12.72)	9.71 (8.03–11.70)	3.96 (3.10–4.83)
Puerto Rico				
Good	64.12 (60.40–67.68)	49.96 (47.28–52.64)	54.60 (52.37–56.80)	52.05 (48.68–55.43)
Moderate	28.24 (24.93–31.82)	41.80 (39.17–44.47)	37.36 (35.24–39.54)	40.26 (36.91–43.61)
Poor	7.63 (5.84–9.92)	8.24 (6.89–9.83)	8.04 (6.93–9.31)	7.68 (6.15–9.22)

^a^Standardised by age, sex and education (U = Urban; R = Rural; Dom Rep = Dominican Republic).

**Table 2.** Mutually adjusted prevalence ratios from a Poisson regression analysis describing the association between poor SRH and age, sex, educational level, number of assets, use of community health services and health factors across sites (U = Urban; R = Rural; Dom Rep = Dominican Republic)

Centre	Age	Sex	Household assets	Educational level	Community health service	Dementia
Cuba	1.00 (1.00–1.01)	0.84 (0.75–0.94)	0.96 (0.91–1.01)	0.98 (0.93–1.03)	1.27 (1.15–1.41)	1.01 (0.85–1.19)
Dom Rep	1.00 (1.00–1.01)	0.91 (0-80-1.03)	0.96 (0.92–1.00)	0.98 (0.92–1.05)	1.31 (1.17–1.48)	1.01 (0.84–1.21)
Peru (U)	1.00 (0.99–1.02)	0.99 (0.82–1.18)	0.90 (0.80–1.01)	0.85 (0.78–0.93)	1.44 (1.21–1.70)	1.15 (0.84–1.57)
Peru (R)	1.01 (0.98–1.02)	1.34 (1.02–1.77)	0.95 (0.86–1.05)	0.90 (0.78–1.05)	1.45 (1.09–1.92)	1.77 (1.14–2.76)
Venezuela	1.01 (1.00–1.02)	0.86 (0.73–1.01)	0.97 (0.91–1.04)	0.99 (0.92–1.08)	1.42 (1.20–1.67)	1.06 (0.80–1.39)
Mexico (U)	0.99 (0.98–1.01)	0.97 (0.81–1.17)	1.01 (0.93–1.09)	0.97 (0.90–1.04)	1.25 (1.01–1.54)	0.96 (0.70–1.32)
Mexico (R)	1.00 (0.99–1.02)	1.04 (0.87–1.25)	0.98 (0.94–1.03)	1.06 (0.95–1.17)	1.19 (0.99–1.44)	1.37 (1.03–1.82)
China (U)	0.99 (0.98–1.01)	0.99 (0.86–1.14)	1.01 (0.92–1.12)	1.00 (0.95–1.05)	1.04 (0.91–1.19)	0.99 (0.72–1.38)
China (R)	1.01 (0.99–1.03)	1.30 (1.02–1.66)	0.92 (0.85–0.99)	0.89 (0.78–1.02)	2.20 (1.60–3.02)	1.25 (0.73–2.15)
India (U)	1.01 (0.99–1.03)	0.63 (0.47–0.84)	0.88 (0.80–0.96)	1.09 (0.96–1.24)	2.07 (1.55–2.77)	1.36 (0.92–2.01)
India (R)	1.02 (1.00–1.03)	1.00 (0.84–1.18)	0.97 (0.92–1.02)	0.91 (0.82–1.02)	1.33 (1.10–1.61)	1.24 (0.99–1.57)
Puerto Rico	1.01 (1.00–1.02)	0.83 (0.72–0.97)	0.99 (0.98–1.10)	0.91 (0.86–0.97)	1.56 (1.26–1.93)	1.12 (0.89–1.41)
Pooled estimate	1.01 (1.00–1.01)	0.93 (0.89–0.98)	0.96 (0.94–0.98)	0.96 (0.94–0.98)	1.32 (1.26–1.39)	1.11 (1.03–1.2)
*I*^2^	23.9%	64.4%	0%	53.6%	70.6%	11.5%
Centre	Stroke	Hypertension	COPD	Diabetes	Depression	Anxiety
Cuba	1.36 (1.16–1.60)	1.09 (0.97–1.23)	1.34 (1.07–1.67)	1.24 (1.11–1.40)	1.68 (1.49–1.89)	1.15 (1.09–1.21)
Dom Rep	1.18 (0.99–1.42)	1.17 (1.01–1.36)	1.47 (1.22–1.76)	1.11 (0.95–1.29)	1.69 (1.48–1.93)	1.17 (1.11–1.23)
Peru (U)	1.36 (1.05–1.76)	1.11 (0.94–1.31)	1.11 (0.82–1.50)	0.98 (0.75–1.29)	1.64 (1.35–1.98)	1.10 (1.03–1.18)
Peru (R)	1.27 (0.71–2.25)	1.51 (1.14–2.01)	1.44 (0.69–3.00)	1.23 (0.82–1.83)	0.97 (0.69–1.36)	1.26 (1.06–1.50)
Venezuela	1.16 (0.93–1.46)	1.20 (0.98–1.47)	1.24 (0.97–1.58)	1.13 (0.95–1.34)	1.77 (1.49–2.10)	1.15 (1.08–1.22)
Mexico (U)	1.30 (0.97–1.73)	1.15 (0.95–1.39)	1.11 (0.80–1.53)	1.04 (0.86–1.26)	1.68 (1.37–2.06)	1.12 (1.02–1.23)
Mexico (R)	1.03 (0.77–1.39)	1.01 (0.85–1.21)	1.38 (1.06–1.79)	1.29 (1.06–1.58)	1.56 (1.28–1.90)	1.19 (1.07–1.31)
China (U)	1.23 (0.98–1.53)	0.97 (0.85–1.11)	0.94 (0.64–1.39)	1.03 (0.87–1.22)	1.31 (0.95–1.82)	0.96 (0.80–1.17)
China (R)	1.27 (0.69–2.34)	3.92 (2.88–5.32)	1.40 (0.77–2.56)	2.14 (1.03–4.42)	2.11 (1.04–4.29)	1.20 (0.83–1.74)
India (U)	3.03 (1.73–5.29)	0.87 (0.66–1.14)	1.52 (0.74–3.13)	1.59 (1.14–2.20)	2.10 (1.59–2.79)	1.30 (1.14–1.48)
India (R)	0.82 (0.30–2.20)	1.06 (0.92–1.22)	1.12 (0.87–1.45)	1.09 (0.82–1.44)	0.98 (0.80–1.21)	1.12 (0.98–1.28)
Puerto Rico	1.30 (1.06–1.60)	1.41 (1.15–1.72)	1.45 (1.09–1.94)	1.46 (1.28–1.66)	1.65 (1.40–1.95)	1.22 (1.13–1.31)
Pooled estimate	1.27 (1.18–1.37)	1.41 (1.09–1.50)	1.29 (1.18–1.40)	1.20 (1.14–1.27)	1.6 (1.51–1.69)	1.16 (1.13–1.18)
*I*^2^	23.1%	86.8%	0%	53.4%	70.7%	14%

### Association between SRH and mortality

The Cox proportional hazards models are presented in Table 3. In the unadjusted model (Model 1), respondents with poor SRH had a 142% increased risk of death within 4 years compared to respondents with moderate SRH (HR = 2.42, 95% CI 1.91–3.07). When adjusting socio-demographic characteristics and use of community health service (Model 2), respondents with poor SRH had a 97% increased risk compared to respondents with moderate SRH (HR = 1.97, 95% CI 1.74–2.23). When adjusting for socio-demographic characteristics, use of community health service, and health factors (Model 3), those with poor SRH had a 61% increase risk compared to respondents with moderate SRH (HR = 1.61, 95% CI 1.40–1.86). In the final model (Model 4), we also adjusted for disability according to WHODAS which showed that those with poor SRH still had a 43% increased risk compared to those with moderate SRH (HR = 1.43, 95% CI 1.23–1.66). The pooled estimates showed that respondents with good SRH had 10% reduction in risk compared to respondents with moderate SRH (HR = 0.90, 95% CI 0.82–0.99).

**Table 3.** The Cox proportional hazards models of the association between SRH and mortality. Moderate SRH used as reference (U = Urban; R = Rural; Dom Rep = Dominican Republic)

	Model 1		Model 2		Model 3		Model 4	
	Poor SRH	Good SRH	Poor SRH	Good SRH	Poor SRH	Good SRH	Poor SRH	Good SRH
Centre								
Cuba	1.38 (1.07–1.78)	0.74 (0.62–0.88)	1.17 (0.91–1.51)	0.69 (0.58–0.82)	1.04 (0.79–1.37)	0.75 (0.62–0.91)	0.94 (0.71–1.26)	0.77 (0.64–0.94)
Dom Rep	2.28 (1.75–2.98)	0.97 (0.79–1.18)	2.30 (1.75–3.02)	1.04 (0.85–1.27)	2.19 (1.63–2.93)	1.07 (0.86–1.32)	1.89 (1.40–2.54)	1.14 (0.92–1.42)
Peru (U)	2.92 (1.64–5.19)	0.66 (0.42–1.01)	2.86 (1.57–5.15)	0.70 (0.45–1.08)	2.45 (1.14–5.24)	0.82 (0.49–1.35)	1.90 (0.80–4.52)	1.19 (0.65–2.17)
Peru (R)	3.66 (1.26–10.59)	0.8 (0.46–1.39)	3.58 (1.21–10.54)	0.90 (0.51–1.60)	3.60 (1.06–12.22)	0.96 (0.52–1.78)	3.96 (1.14–13.71)	0.96 (0.51–1.80)
Venezuela	3.34 (2.17–5.16)	0.6 (0.44–0.82)	3.00 (1.93–4.66)	0.59 (0.43–0.81)	2.70 (1.63–4.46)	0.75 (0.53–1.07)	2.49 (1.42–4.36)	0.79 (0.54–1.15)
Mexico (U)	2.34 (1.30–4.21)	1.01 (0.66–1.56)	2.07 (1.14–3.78)	0.92 (0.60–1.43)	1.94 (1.02–3.70)	0.91 (0.57–1.46)	1.71 (0.88–3.35)	0.98 (0.61–1.56)
Mexico (R)	1.89 (1.11–3.23)	0.72 (0.48–1.09)	1.84 (1.07–3.16)	0.71 (0.47–1.07)	1.58 (0.88–2.84)	0.79 (0.51–1.23)	1.31 (0.69–2.47)	0.80 (0.52–1.25)
China (U)	4.12 (2.64–6.44)	1.09 (0.76–1.55)	2.69 (1.70–4.24)	0.81 (0.56–1.15)	1.76 (0.85–3.64)	0.73 (0.49–1.07)	1.39 (0.65–2.98)	0.75 (0.51–1.10)
China (R)	3.27 (2.14–4.99)	0.94 (0.72–1.22)	2.72 (1.77–4.16)	0.87 (0.66–1.14)	1.90 (1.15–3.13)	1.01 (0.75–1.37)	1.87 (1.08–3.24)	1.03 (0.76–1.41)
India (U)	2.38 (1.23–4.62)	0.92 (0.60–1.40)	2.62 (1.32–5.19)	0.85 (0.56–1.30)	1.97 (0.91–4.26)	1.00 (0.64–1.57)	2.03 (0.91–4.49)	1.22 (0.75–1.98)
India (R)								
Puerto Rico	1.68 (1.18–2.38)	0.67 (0.52–0.86)	1.62 (1.14–2.31)	0.74 (0.58–0.96)	0.84 (0.51–1.40)	0.75 (0.56–1.01)	0.73 (0.43–1.23)	0.78 (0.58–1.05)
Pooled estimate	2.42 (1.91–3.07)	0.81 (0.74–0.88)	1.97 (1.74–2.23)	0.79 (0.72–0.86)	1.61 (1.40–1.86)	0.86 (0.78–0.94)	1.43 (1.23–1.66)	0.90 (0.82–0.99)
*I*^2^	68.7%	38.6%	66.2%	32.9%	64.6%	0%	62.5%	21.9%

## Discussion

This study aimed to explore the prevalence of SRH and its association with mortality in older adults from China, India and Latin America in order to cross-culturally validate the construct of SRH. In comparison with older adults rating their health as moderate, those rating it as poor had a 142% increased risk of dying within 4 years. After controlling for socio-demographic characteristics, use of community health service, health factors and disability, those with poor SRH had a 43% increased risk compared to individuals assessing their health as moderate. We also found that individuals rating their health as good had a 10% reduction in risk compared to individuals assessing their health as moderate. This consistent association between SRH and mortality is in accordance with findings from high-income countries [3, 4], and our findings support the use of SRH as a simple measure in survey settings to identify vulnerable groups and to evaluate health interventions in resource-scares settings. As a person may be intuitively aware of pathologic processes before they become measurable, SRH could be a valuable instrument for identifying older adults at risk [32–34]. Studies suggests that SRH may act as a proxy for other covariates that are known to predict health [1, 3]; it may reflect experiential knowledge grounded in both bodily experience and social interaction [35, 36]; it may be associated with serum inflammatory markers [37], or it may reflect perceived declines in health, rather than current health levels [2–4]. In addition, previous and present health experiences are likely to influence both the range of factors that a person reviews as potential components of SRH, and the way in which they are taken into account [38].

The analyses in this study were conducted on large population-based samples, hence allowing us to assess the consistency or cultural specificity of the observed associations. Diverse cultural patterns of experiencing and reporting illness and health may have several origins that call for critical scrutiny taking note of positional perspectives [39, 40], and the general morbidity in a population may influence the understanding of which symptoms that warrant attention [6, 8]. Age-related changes in coping suggests that different age-groups may act differently in response to chronic symptoms, since older adults may attribute them to ageing rather than to illness, and people living in different cultures may vary in their willingness to present positive or negative pictures of themselves [41].

The strengths of this study include the use of a large, population-based sample with over 50,000 person-years of follow-up. To our knowledge, this is the first international study using standardised questionnaires to assess the relationship between SRH and mortality across a wide range of cultures in LMIC. However, some limitations deserve mentioning. Every effort was made to ensure conceptual equivalence of all items included in study instruments. In addition to translation and back translation procedures, all teams underwent substantial training to ensure a consistent approach in the administration of measures across settings, in accordance with manualised standard operational procedures. However, cross-cultural differences in understandings cannot be eliminated. The extent to which there were variations in comprehension of the SRH question is unclear. Our findings may not be generalisable beyond the particular catchment area sites where the study was carried out, and should not be taken to refer to the respective countries as a whole. In this study, catchment areas selected were as representative as possible of the wider geographical region. For urban catchment areas, predominantly middle-class or professional areas with high-income earners were avoided. Rural areas were defined by low population density and traditional agrarian lifestyle.

SRH was assessed at baseline in late life, with no information regarding either exposure earlier in the life course, or subsequent changes in SRH. We acknowledge the potential risk of over adjustment, since age, sex, household assets, educational level, use of community health services, health factors and disability, may mediate the association between SRH and mortality. Potentially important factors such as social capital, activity engagement and sense of community are likely to have influenced the association between SRH and mortality. However, these measures were not available in the dataset used. There may have been some potential confounders, such as personality type [42] that we could not adjust for due to lack of data. Lastly, we did not include cause of death in our analyses and the predictive power of SRH for cause-specific mortality might have varied according to cause of death.

Given the growing number of older adults in LMIC, the rising importance of non-communicable diseases and the scarcity of studies on SRH in older adults in these regions, our findings may have implications for a better understanding of the association between SRH and mortality. Our findings may also support the use of SRH as a simple measure in survey settings to identify vulnerable groups of older adults and evaluate health interventions in resource-scares settings.

**Key points**

- SRH is useful in LMIC settings as a brief and simple measure of overall subjective health status.
- SRH might add an important dimension to objective measures of function to detect those at risk of developing dependence.
- SRH can help identify vulnerable groups of older people in resource-scarce settings for targeted health interventions.

## Supplementary Material

Supplementary Data

Supplementary Data

Supplementary Data

Supplementary Data
