# Supplementary material for: Self-rated health and its association with mortality in older adults in China, India and Latin America—a 10/66 Dementia Research Group study
Source: Age Ageing. 2017 Jul 18;46(6):932–9. doi: 10.1093/ageing/afx126 (PMC5860352; doi:10.1093/ageing/afx126)
Supplement: Supplementary Data [file afx126_aa-16-0903-file006.docx]

|  | **Cuba** | **Dom Rep** | **Peru (U)** | **Peru (R)** | **Venezuela** | **Mexico (U)** | **Mexico (R)** | **China (U)** | **China (R)** | **India (U)** | **India (R)** | **Puerto Rico** | **TOTAL** |
| --- | --- | --- | --- | --- | --- | --- | --- | --- | --- | --- | --- | --- | --- |
|  | n=2928 (%) | n=2009 (%) | n=1373 (%) | n=551 (%) | n=1916 (%) | n=1001 (%) | n=1000 (%) | n=1160 (%) | n=1002 (%) | n=999 (%) | n=999 (%) | n=2002 (%) | n=16940 (%) |
| **Age, n (%)** |  |  |  |  |  |  |  |  |  |  |  |  |  |
| 65-69 | 758 (25.89) | 533 (26.53) | 373 (27.17) | 179 (32.49) | 830 (43.32) | 245 (24.48) | 299 (29.90) | 316 (27.24) | 383 (38.22) | 413 (41.34) | 331 (33.13) | 411 (20.53) | 5071 (31.81) |
| 70-74 | 788 (26.91) | 519 (25.83) | 352 (25.64) | 140 (25.41) | 461 (24.06) | 329 (32.87) | 252 (25.20) | 362 (31.21) | 296 (29.45) | 318 (31.83) | 350 (35.04) | 455 (22.73) | 4622 (29.00) |
| 75-79 | 637 (21.76) | 397 (19.76) | 296 (21.56) | 101 (18.33) | 335 (17.48) | 204 (20.38) | 221 (22.10) | 254 (21.90) | 202 (20.16) | 144 (14.41) | 177 (17.72) | 483 (24.13) | 3451 (21.65) |
| ≥80 | 745 (25.44) | 560 (27.87) | 352 (25.64) | 131 (23.77) | 290 (15.14) | 223 (22.28) | 228 (22.80) | 228 (19.66) | 121 (12.08) | 124 (12.41) | 141 (14.11) | 653 (32.62) | 3796 (23.81) |
| *Missing values* | 7 (0.24) | 0 (0) | 1 (0.07) | 0 (0) | 3 (0.16) | 1 (0.10) | 0 (0) | 0 (0) | 0 (0) | 4 (0.40) | 0 (0) | 0 (0) | 16 (1.00) |
| **Women, n (%)** | 1907 (64.97) | 1325 (65.99) | 885 (64.41) | 295 (53.36) | 1217 (63.42) | 665 (66.37) | 602 (60.20) | 661 (56.98) | 556 (55.49) | 571 (57.68) | 545 (54.55) | 1347 (67.28) | 10576 (66.35) |
| *Missing values* | 0 (0) | 1 (0.05) | 0 (0) | 0 (0) | 0 (0) | 0 (0) | 0 (0) | 0 (0) | 0 (0) | 13 (1.30) | 0 (0) | 0 (0) | 14 (0.88) |
| **Household assets, mean (SD)** | 5.67 (0.97) | 4.98 (1.43) | 6.14 (0.60) | 4.70 (1.33) | 6.19 (0.99) | 6.05 (1.03) | 4.05 (1.76) | 5.50 (0.66) | 5.60 (1.37) | 4.12 (1.54) | 2.77 (1.52) | 6.69 (0.59) | 5.42 (1.52) |
| **Education, n (%)** |  |  |  |  |  |  |  |  |  |  |  |  |  |
| None | 75 (2.56) | 392 (19.51) | 37 (2.69) | 84 (15.25) | 153 (7.97) | 227 (22.65) | 327 (32.70) | 232 (20.00) | 579 (57.78) | 428 (42.67) | 660 (66.70) | 72 (3.60) | 3266 (20.50) |
| Some, but did not complete primary | 655 (22.32) | 1022 (50.87) | 90 (6.55) | 141 (25.59) | 443 (23.08) | 353 (35.23) | 510 (51.00) | 153 (13.19) | 114 (11.38) | 234 (23.33) | 195 (19.52) | 389 (19.43) | 4299 (26.97) |
| Completed Primary | 978 (33.32) | 370 (18.42) | 458 (33.33) | 267 (48.46) | 958 (49.92) | 229 (22.85) | 122 (12.20) | 303 (26.12) | 259 (25.85) | 211 (21.04) | 116 (11.61) | 415 (20.73) | 4686 (29.40) |
| Completed Secondary (metric) | 727 (24.77) | 135 (6.72) | 479 (34.86) | 36 (6.53) | 266 (13.86) | 99 (9.88) | 25 (2.50) | 335 (28.88) | 45 (4.49) | 87 (8.67) | 26 (2.60) | 713 (35.61) | 2973 (18.65) |
| Completed Tertiary (college) | 499 (17.00) | 73 (3.63) | 305 (22.20) | 16 (2.90) | 93 (4.85) | 92 (9.18) | 16 (1.60) | 137 (11.81) | 5 (0.50) | 42 (4.19) | 2 (0.20) | 410 (20.48) | 1690 (10.60) |
| *Missing values* | 1 (0.03) | 17 (0.85) | 5 (0.36) | 7 (1.27) | 6 (0.31) | 2 (0.20) | 0 (0) | 0 (0) | 0 (0) | 1 (0.10) | 0 (0) | 3 (0.15) | 42 (0.26) |
| **Use of CHS last 3 months, n (%)** |  |  |  |  |  |  |  |  |  |  |  |  |  |
| None | 1471 (50.12) | 1049 (52.22) | 705 (51.31) | 396 (71.87) | 712 (37.10) | 281 (28.04) | 354 (35.40) | 712 (61.38) | 941 (93.91) | 437 (43.57) | 322 (32.23) | 363 (18.13) | 7743 (48.58) |
| ≥1 services used | 1464 (49.88) | 960 (47.78) | 669 (48.69) | 155 (28.13) | 1207 (62.90) | 721 (71.96) | 646 (64.60) | 448 (38.62) | 61 (6.09) | 566 (56.43) | 677 (67.77) | 1639 (81.87) | 9213 (57.80) |
| *Missing values* | 0 (0) | 0 (0) | 0 (0) | 0 (0) | 0 (0) | 0 (0) | 0 (0) | 0 (0) | 0 (0) | 0 (0) | 0 (0) | 0 (0) | 0 (0) |
| **Supplementary Table 1.** Socio-demographic characteristics by study site (U= Urban; R= Rural; Dom Rep= Dominican Republic; CHS= Community Health Services). | | | | | | | | | | | | |  |
