# Supplementary material for: Self-rated health and its association with mortality in older adults in China, India and Latin America—a 10/66 Dementia Research Group study
Source: Age Ageing. 2017 Jul 18;46(6):932–9. doi: 10.1093/ageing/afx126 (PMC5860352; doi:10.1093/ageing/afx126)
Supplement: Supplementary Data [file afx126_aa-16-0903-file007.docx]

|  | **Very good** | **Good** | **Moderate** | **Poor** | **Very Poor** |
| --- | --- | --- | --- | --- | --- |
| **Cuba** | 10.26 (9.19-11.43) | 42.59 (40.80-44.40) | 37.92 (36.18-39.70) | 7.77 (6.85-8.80) | 1.47 (1.08-1.98) |
| **Dom Rep** | 13.54 (12.03-15.20) | 34.80 (32.72-36.93) | 42.41 (40.21-44.64) | 7.22 (6.15-8.45) | 2.04 (1.51-2.76) |
| **Peru (U)** | 19.87 (17.69-22.24) | 36.17 (33.63-38.80) | 38.86 (36.26-41.54) | 4.08 (3.13-5.29) | 1.02 (0.61-1.71) |
| **Peru (R)** | 24.68 (21.09-28.66) | 34.48 (30.50-38.70) | 38.84 (34.68-43.16) | 1.81 (0.98-3.35) | 0.18 (0.03-1.28) |
| **Venezuela** | 15.00 (13.37-16.80) | 44.14 (41.85-46.45) | 36.32 (34.13-38.57) | 3.65 (2.89-4.60) | 0.89 (0.55-1.42) |
| **Mexico (U)** | 20.86 (18.46-23.48) | 30.94 (28.07-33.96) | 39.52 (36.55-42.57) | 7.09 (5.65-8.86) | 1.60 (0.96-2.65) |
| **Mexico (R)** | 18.30 (15.97-20.88) | 32.90 (30.01-35.93) | 40.40 (37.30-43.58) | 7.10 (5.66-8.87) | 1.30 (0.76-2.22) |
| **China (U)** | 2.07 (1.37-3.11) | 13.10 (11.15-15.34) | 81.21 (78.70-83.48) | 3.02 (2.18-4.16) | 0.60 (0.29-1.26) |
| **China (R)** | 24.85 (21.99-27.95) | 44.02 (40.70-47.40) | 26.95 (24.11-29.98) | 2.99 (2.11-4.24) | 1.20 (0.68-2.09) |
| **India (U)** | 41.66 (38.55-44.87) | 36.60 (33.62-39.67) | 17.15 (14.94-19.61) | 3.99 (2.94-5.40) | 0.60 (0.27-1.32) |
| **India (R)** | 23.02 (14.85-35.54) | 23.12 (20.59-25.87) | 64.86 (61.88-67.74) | 8.01 (6.48-9.86) | 1.70 (1.06-2.72) |
| **Puerto Rico** | 7.84 (6.73-9.12) | 46.75 (44.54-48.98) | 37.36 (35.24-39.54) | 6.19 (5.22-7.34) | 1.85 (1.34-2.54) |
| **Supplementary Table 2.** Prevalence ratios (95% CI) of self-rated health (SRH) by site with the five levels of SRH as measured in the original survey (U= Urban; R= Rural; Dom Rep= Dominican Republic). | | | | | |
