# Supplementary material for: Self-rated health and its association with mortality in older adults in China, India and Latin America—a 10/66 Dementia Research Group study
Source: Age Ageing. 2017 Jul 18;46(6):932–9. doi: 10.1093/ageing/afx126 (PMC5860352; doi:10.1093/ageing/afx126)
Supplement: Supplementary Data [file afx126_aa-16-0903-file008.docx]

|  | **65-69 years** | **70-74 years** | **75-79 years** | **≥80 years** |
| --- | --- | --- | --- | --- |
| **Cuba** |  |  |  |  |
| *Good* | 51.58 (47.96-55.19) | 54.82 (51.35-58.25) | 52.12 (48.25-55.97) | 52.48 (48.86-56.08) |
| *Moderate* | 39.97 (36.50-43.55) | 36.29 (33.01-39.71) | 39.25 (35.53-43.10) | 36.51 (33.10-40.06) |
| *Poor* | 8.44 (6.64-10.68) | 8.88 (7.10-11.07) | 8.63 (6.71-11.05) | 11.01 (8.96-13.46) |
| **Dom Rep** |  |  |  |  |
| *Good* | 51.59 (47.29-55.88) | 51.10 (45.75-54.45) | 47.61 (42.73-52.53) | 44.11 (40.03-48.26) |
| *Moderate* | 39.96 (35.85-44.22) | 39.88 (35.72-44.20) | 44.58 (39.77-49.50) | 45.54 (41.42-49.72) |
| *Poor* | 8.44 (6.37-11.11) | 10.02 (7.72-12.91) | 7.81 (5.55-10.88) | 10.36 (8.10-13.15) |
| **Peru (U)** |  |  |  |  |
| *Good* | 62.73 (57.63-67.57) | 57.10 (52.00-62.05) | 53.72 (47.98-59.36) | 50.00 (44.61-55.39) |
| *Moderate* | 32.98 (28.32-37.99) | 38.64 (33.77-43.74) | 40.54 (35.00-46.33) | 43.75 (38.47-49.18) |
| *Poor* | 4.29 (2.64-6.89) | 4.26 (2.59-6.94) | 5.74 (3.60-9.03) | 6.25 (4.17-9.25) |
| **Peru (R)** |  |  |  |  |
| *Good* | 58.10 (40.48-65.35) | 61.43 (52.86-69.34) | 65.35 (55.68-73.89) | 53.44 (44.94-61.73) |
| *Moderate* | 40.78 (33.58-48.40) | 37.14 (29.33-45.69) | 30.69 (22.56-40.24) | 44.27 (36.08-52.79) |
| *Poor* | 1.12 (0.28-4.36) | 1.43 (0.36-5.54) | 3.96 (1.49-10.09) | 2.29 (0.74-6.87) |
| **Venezuela** |  |  |  |  |
| *Good* | 62.65 (59.22-65.96) | 60.30 (55.73-64.71) | 56.12 (50.62-61.48) | 50.69 (44.89-56.47) |
| *Moderate* | 34.34 (31.10-37.73) | 36.44 (32.15-40.96) | 37.91 (32.74-43.37) | 40.00 (34.46-45.81) |
| *Poor* | 3.01 (2.01-4.48) | 3.25 (1.97-5.33) | 5.97 (3.88-9.08) | 9.31 (6.46-13.25) |
| **Mexico (U)** |  |  |  |  |
| *Good* | 52.24 (45.75-58.66) | 50.46 (45.13-55.77) | 50.00 (43.38-56.62) | 55.16 (48.56-61.58) |
| *Moderate* | 40.40 (33.96-46.36) | 40.73 (35.55-46.12) | 41.18 (34.60-48.08) | 35.87 (29.95-42.27) |
| *Poor* | 7.76 (5.02-11.80) | 8.81 (6.16-12.46) | 8.82 (5.67-13.48) | 8.97 (5.89-13.42) |
| **Mexico (R)** |  |  |  |  |
| *Good* | 50.84 (45.17-56.48) | 54.37 (48.14-60.45) | 50.68 (44.08-57.25) | 48.68 (42.27-55.14) |
| *Moderate* | 40.47 (35.00-46.18) | 40.87 (34.87-47.17) | 39.82 (33.53-46.46) | 40.35 (34.02-47.02) |
| *Poor* | 8.70 (5.99-12.46) | 4.76 (2.73-8.18) | 9.50 (6.30-14.08) | 10.96 (7.58-15.61) |
| **China (U)** |  |  |  |  |
| *Good* | 12.66 (9.36-16.90) | 12.15 (9.03-16.17) | 16.54 (12.43-21.66) | 21.93 (16.78-28.13) |
| *Moderate* | 84.81 (80.35-88.40) | 84.81 (80.57-88.25) | 80.31 (74.99-84.74) | 71.49 (65.07-77.15) |
| *Poor* | 2.53 (1.28-4.96) | 3.04 (1.69-5.39) | 3.15 (1.59-6.15) | 6.58 (4.03-10.56) |
| **China (R)** |  |  |  |  |
| *Good* | 71.02 (65.90-75.65) | 64.86 (58.92-70.38) | 68.32 (61.49-74.44) | 72.73 (64.13-79.91) |
| *Moderate* | 27.15 (22.67-32.16) | 30.41 (25.21-36.15) | 26.24 (20.43-33.01) | 19.01 (13.00-26.93) |
| *Poor* | 1.83 (0.88-3.77) | 4.73 (2.83-7.81) | 5.45 (3.06-9.51) | 8.26 (4.49-14.73) |
| **India (U)** |  |  |  |  |
| *Good* | 79.18 (74.97-82.84) | 81.13 (76.44-85.07) | 75.00 (67.33-81.37) | 70.97 (62.21-78.40) |
| *Moderate* | 16.95 (13.62-20.89) | 13.21 (9.90-17.40) | 21.53 (15.59-28.95) | 23.39 (16.61-31.87) |
| *Poor* | 3.87 (2.39-6.23) | 5.66 (3.59-8.81) | 3.47 (1.45-8.08) | 5.65 (2.71-11.38) |
| **India (R)** |  |  |  |  |
| *Good* | 35.65 (30.64-40.99) | 21.29 (20.13-28.99) | 17.51 (12.61-23.80) | 14.18 (9.33-20.98) |
| *Moderate* | 58.01 (52.59-63.23) | 67.14 (62.13-71.79) | 70.62 (63.52-76.85) | 68.09 (60.01-75.20) |
| *Poor* | 6.34 (4.17-9.54) | 8.57 (6.06-11.99) | 11.86 (7.86-17.53) | 17.73 (12.30-24.87) |
| **Puerto Rico** |  |  |  |  |
| *Good* | 56.45 (51.50-61.27) | 60.00 (55.36-64.47) | 56.52 (52.06-60.88) | 48.24 (44.39-52.11) |
| *Moderate* | 35.04 (30.51-39.84) | 33.85 (29.56-38.41) | 36.44 (32.26-40.83) | 41.96 (38.25-45.76) |
| *Poor* | 8.52 (6.18-11.62) | 6.15 (4.23-8.86) | 7.04 (5.08-9.68) | 9.80 (7.79-12.26) |
| **Supplementary Table 3.** Prevalence ratios (95% CI) of self-rated health (SRH) by age group and site. * Standardized by age, sex, and educational level (U= Urban; R= Rural; Dom Rep= Dominican Republic). | | | | |
