# Supplementary material for: Self-rated health and its association with mortality in older adults in China, India and Latin America—a 10/66 Dementia Research Group study
Source: Age Ageing. 2017 Jul 18;46(6):932–9. doi: 10.1093/ageing/afx126 (PMC5860352; doi:10.1093/ageing/afx126)
Supplement: Supplementary Data [file afx126_aa-16-0903-file009.docx]

Supplementary data, Appendix 1

The title of the 10/66 Dementia Research Group (DRG) reflects the fact that, when the group was formed in 1998, less than 10% of population-based research on dementia had been carried out in developing countries although 66% of those affected lived in those settings [21]. The 10/66 DRG research program was developed to address this inequity, quantifying dementia prevalence, incidence and impact across Latin American countries, China and India, using a validated methodology. However, given that this was a population cohort, the scope of the research was much broader than this—entailing a comprehensive enquiry into health and social aspects of ageing [19].
